# GmGLU1 and GmRR4 contribute to iron deficiency tolerance in soybean

**DOI:** 10.3389/fpls.2024.1295952

**Published:** 2024-02-27

**Authors:** Daniel R. Kohlhase, Jamie A. O’Rourke, Michelle A. Graham

**Affiliations:** ^1^ Department of Agronomy, Iowa State University, Ames, IA, United States; ^2^ United States Department of Agriculture, Agricultural Research Service, Corn Insects and Crop Genetics Research Unit and Department of Agronomy, Iowa State University, Ames, IA, United States

**Keywords:** *Glycine max*, soybean, virus-induced gene silencing, RR4, bHLH38, GLU1, iron deficiency

## Abstract

Iron deficiency chlorosis (IDC) is a form of abiotic stress that negatively impacts soybean yield. In a previous study, we demonstrated that the historical IDC quantitative trait locus (QTL) on soybean chromosome Gm03 was composed of four distinct linkage blocks, each containing candidate genes for IDC tolerance. Here, we take advantage of virus-induced gene silencing (VIGS) to validate the function of three high-priority candidate genes, each corresponding to a different linkage block in the Gm03 IDC QTL. We built three single-gene constructs to target *GmGLU1* (*GLUTAMATE SYNTHASE 1*, Glyma.03G128300), *GmRR4* (*RESPONSE REGULATOR 4*, Glyma.03G130000), and *GmbHLH38* (*beta Helix Loop Helix 38*, Glyma.03G130400 and Glyma.03G130600). Given the polygenic nature of the iron stress tolerance trait, we also silenced the genes in combination. We built two constructs targeting *GmRR4*+*GmGLU1* and *GmbHLH38*+*GmGLU1*. All constructs were tested on the iron-efficient soybean genotype Clark grown in iron-sufficient conditions. We observed significant decreases in soil plant analysis development (SPAD) measurements using the *GmGLU1* construct and both double constructs, with potential additive effects in the *GmRR4*+*GmGLU1* construct. Whole genome expression analyses (RNA-seq) revealed a wide range of affected processes including known iron stress responses, defense and hormone signaling, photosynthesis, and cell wall structure. These findings highlight the importance of GmGLU1 in soybean iron stress responses and provide evidence that IDC is truly a polygenic trait, with multiple genes within the QTL contributing to IDC tolerance. Finally, we conducted BLAST analyses to demonstrate that the Gm03 IDC QTL is syntenic across a broad range of plant species.

## 1 Introduction

In the market year 2021/2022, the United States produced 4.47 billion bushels of soybean (*Glycine max* (L.) Merrill), valued at $59.2 billion and accounting for almost 60% of world oilseed production^1^. High yields are essential to soybean profitability, while diseases, pests, and abiotic stress negatively impact soybean yield. In the northern Midwest United States, a major soybean production area, high moisture, high pH (>7.2), and calcareous soils limit iron availability and uptake and promote the development of iron deficiency chlorosis (IDC; Hansen et al., 2003; Merry et al., 2022). In a survey by Hansen et al. (2003), Minnesota soybean producers estimated that 24% of the soybean crop was impacted by IDC. A corresponding field survey confirmed that 22% of each field (on average) was severely impacted by IDC. Froechlich and Fehr (1981) found a 20% reduction in yield for every point on the standard five-point IDC rating system. Similarly, Kaiser et al. (2014) found that chlorosis rating scores >2.5 resulted in relative yield loss >35% when comparing susceptible and tolerant varieties with no IDC management. The best management recommendation to prevent IDC-related yield loss is to plant iron-efficient soybean varieties (Kaiser et al., 2014; Merry et al., 2022). However, these lines do not perform as well as elite lines when IDC conditions are not present. To close the yield gap and reduce IDC-related yield loss, it is imperative that we continue to study the genetics of iron-efficient lines.

Various approaches, including association mapping and gene expression studies, have been used to study soybean responses to IDC. Association mapping and genome-wide association (GWA) studies have identified various quantitative trait loci (QTLs) associated with iron efficiency. Cianzio (1980) initially suggested that the soybean IDC response is controlled by a single major locus. Later, Lin et al. (1997) used two mapping populations to study the inheritance of iron efficiency. In one population, Anoka × A7, a single major locus was identified, accounting for 68.8% to 72.7% of response variation. In the second population, Pride B216 × A15, multiple loci with smaller effects were identified. Merry et al. (2019) used a GWA study and fine mapping of a population developed from Fiskeby III × Mandarin [Ottawa] to identify three QTLs associated with IDC tolerance. While each of the previous studies had identified the historical IDC QTL on chromosome Gm03, Merry et al. (2019) also identified a novel QTL on Gm05. Assefa et al. (2020) used 460 soybean accessions from 27 countries in a GWA study to identify 69 regions of interest, including the QTL on Gm03. Further linkage disequilibrium analysis revealed four major linkage blocks, suggesting that multiple genes are involved in the soybean IDC response.

Gene expression studies have helped to identify many genes that are differentially expressed in response to iron stress. O’Rourke et al. (2009) and Atencio et al. (2021) used the near-isogenic lines Clark (IDC tolerant) and IsoClark (IDC susceptible) to characterize iron stress responses at 2, 10, and 14 days after iron stress. Moran Lauter et al. utilized RNA-seq to study the early transcriptional responses in Clark leaves and roots at 1 hour and 6 hours after iron stress (Moran Lauter et al., 2014) and 30, 60, and 120 minutes after iron stress (Moran Lauter et al., 2020). Collectively, these studies identified the hallmarks of the iron stress response, regulation of genes involved in defense and stress, iron homeostasis, and DNA replication/methylation in Clark. Kohlhase et al. (2021) conducted RNA-seq analyses of leaves and roots from nine IDC-tolerant and nine IDC-susceptible lines (members of the Assefa et al. (2020) GWA panel) at 1 hour after iron stress. Little overlap in differentially expressed genes (DEGs) was found between the nine IDC-tolerant lines, confirming that multiple IDC tolerance responses are present within the soybean germplasm collection.

While scientists have identified genomic regions of interest and genes that respond to iron stress, validation of candidate genes *via* genetic transformation continues to present a bottleneck in soybean (Xu et al., 2022). In soybean, researchers have adopted virus-induced gene silencing (VIGS) as a relatively fast and inexpensive tool that can target single genes or gene families (Zhang and Ghabrial, 2006; Zhang et al., 2013). Targeted traits include resistance genes and defense gene networks (Meyer et al., 2009; Liu et al., 2011; Pandey et al., 2011; Liu et al., 2012; Cooper et al., 2013; Kandoth et al., 2013; Morales et al., 2013; Xu et al., 2018; Pedley et al., 2019) and candidate abiotic stress genes (Atwood et al., 2014; Sun et al., 2016; Ogata et al., 2017; O’Rourke et al., 2021; O’Rourke and Graham, 2022). Atwood et al. (2014) used VIGS to silence *Replication Protein A subunit 3* (*GmRPA3c*), one of the most significantly differentially expressed genes identified by O’Rourke et al. (2009), which is located within an IDC QTL on soybean chromosome Gm20 (Lin et al., 1997, Lin et al., 1998). Silencing *GmRPA3c* in the IDC-susceptible line IsoClark, to mirror its expression in IDC-tolerant Clark, resulted in improved IDC symptoms. RNA-seq of *GmRPA3c*-silenced plant and empty vector controls revealed that *GmRPA3c* silencing resulted in massive transcriptional reprogramming of genes associated with defense, immunity, aging, death, protein modification, protein synthesis, photosynthesis, and iron uptake and transport (Atwood et al., 2014). Similarly, O’Rourke et al. (2021) used VIGS to target 10 genes within the IDC QTL on Gm05 identified by Merry et al. (2019). Silencing a MATE transporter (*Glyma.05G001400*) resulted in increased IDC symptoms in iron-sufficient conditions and differential expression of genes involved in phosphate homeostasis.

In this study, we used single and double VIGS constructs coupled with RNA-seq analyses to target multiple genes in the IDC QTL on soybean chromosome Gm03. Among the 58 candidate genes identified by Assefa et al. (2020), we focused on genes with homology to *Arabidopsis* genes *AtGLU1* (*Glutamate synthase 1*, *GmGLU1*, Glyma.03G128300), At*RR4* (*Response regulator 4*, *GmRR4*, Glyma.03G130000), and *GmbHLH38* (Glyma.03G130400 and Glyma.03G130600, tandem duplicates), representing three of the four linkage groups identified by Assefa et al. (2020). In *Arabidopsis*, an *AtGLU1* mutant has been found to exhibit chlorosis symptoms in low iron conditions, along with reduced expression of iron stress-responsive genes *AtFIT*, *AtFRO2*, and *AtIRT1* in the roots and *bHLH38*, *bHLH39*, *bHLH100*, and *bHLH101* in the shoots (Cui et al., 2020). AtRR4 (also known as ARR9) is regulated by the circadian clock and by cytokinin (Ishida et al., 2008). Li et al. (2019) demonstrated that iron stress targets circadian clock components. GmbHLH38s (Glyma.03G130400 and Glyma.03G130600) were identified by Peiffer et al. (2012) as the most likely genes underlying the IDC QTL on soybean chromosome Gm03. In *Arabidopsis*, bHLH38 and bHLH39 interact with FIT to regulate the expression of iron uptake genes in the root (Yuan et al., 2008). In previous studies, *GmGLU1* and Glyma.03G130400 (*GmbHLH38*) have been found to be repressed by iron stress in Clark roots 30 minutes after exposure to iron stress (Moran Lauter et al., 2020). Conversely, *GmRR4* and Glyma.03G130600 (*GmbHLH38*) were found to be induced by iron stress in Clark roots 30 minutes after exposure to iron stress (Moran Lauter et al., 2020). Glyma.03G130600 (*GmbHLH38*) was also found to be induced by iron stress in Clark roots 1 hour after exposure to iron stress (Moran Lauter et al., 2014). In addition to being differentially expressed in response to iron stress, Peiffer et al. (2012) found a 12-base pair deletion in Glyma.03G130400 associated with iron-inefficient cultivars. While multiple VIGS constructs have targeted Glyma.03G130400 and Glyma.03G130600, none have resulted in significant phenotypic changes (Assefa et al., 2020). Therefore, the objective of this study was to use VIGS coupled with RNA-seq to examine the roles of *GmGLU1*, *GmRR4*, and *GmbHLH38* on iron stress tolerance in soybean. Identifying the genes and networks underlying the IDC QTL on Gm03 will aid in the development of soybean cultivars with improved iron stress tolerance.

## 2 Materials and methods

### 2.1 Virus-induced gene silencing of candidate IDC tolerance genes

The soybean genome sequence of cultivar William 82 (Schmutz et al., 2010; *G. max* Wm82.a2.v1, Phytozome 12, 6/27/2018) was used to design primers for VIGS construct development. Primers were designed (**Supplementary File 1**) using the coding sequence for three candidate genes of interest from the IDC QTL on Gm03: *GmGLU1* (Glyma.03G128300), *GmRR4* (Glyma.03G130000), and *GmbHLH38* (Glyma.03G130400 and Glyma.03G130600). Since soybean has a duplicated genome (Schmutz et al., 2010), we intentionally designed the VIGS constructs to downregulate both the target and homeologous genes. Primers were used to amplify candidate genes from Williams 82 genomic DNA. *Xho*I and *Bam*HI restriction sites were included in the primer sequences to facilitate directional cloning into RNA2 of the *bean pod mottle virus* (BPMV) IA-1033 VIGS vector as described by Whitham et al. (2016). Williams 82 has been described as resistant (Charlson et al., 2004) or moderately tolerant (Witt and Schapaugh, 1995) to IDC. Previous studies have demonstrated the feasibility of using the Williams 82 genome sequence to silence candidate IDC genes in different soybean genotypes (Atwood et al., 2014; O’Rourke and Graham, 2022).

In addition to building single-gene constructs, we also created two constructs that would target two genes simultaneously: *GmRR4* with *GmGLU1* and *GmbHLH38* with *GmGLU1*. The target sequences of the double constructs were identical to those of the individual constructs. To develop each double construct, we needed two primer pairs (**Supplementary File 1**) that would amplify the target sequences for the first and second genes of interest. For amplification of the target sequence of the first gene, we used the same 5′ primer used for the single construct. The 3′ primer was approximately 40 bp long and overlapped the 3′ end of the first gene target sequence by approximately 20 bases and the 5′ end of the second gene target sequence by approximately 20 bases. For amplification of the target sequence of the second gene, the 5′ primer corresponded to the opposite strand of the 3′ primer for gene 1. The second primer for the target sequence of gene 2 was the 3′ primer used to develop the single-gene construct. The gene fragments for each target gene were amplified individually with Invitrogen™ Platinum™ *Taq* DNA Polymerase High Fidelity (Thermo Fisher Scientific, Waltham, MA, USA) in 50-µl reactions. The products were cleaned using the QIAquick PCR Purification kit (Qiagen, Germantown, MD, USA), eluted in 30 µl of sterile, nuclease-free water, and quantified using an ND-1000 NanoDrop™ Spectrophotometer (Thermo Fisher Scientific, Waltham, MA, USA). The two amplification products were then ligated together using the Gibson Assembly Master Mix (New England Biolabs, Inc., Ipswich, MA, USA) protocol (Gibson et al., 2009) and cloned into the BMPV vector following the Whitham protocol (Whitham et al., 2016).

The orientation and identity of the VIGS inserts (**Supplementary File 1**) were confirmed by sequencing using vector-specific primers BPMV_IA1033_MCS_F CTACAGTTTTTGACATTCTCC and BPMV_IA1033_MCS_R ATAGACAGAGCATACTCAACG and the Applied Biosystems™ BigDye™ v3.1 chemistry protocol (Thermo Fisher Scientific, Waltham, MA, USA) with Hi-Di™ Formamide (Thermo Fisher Scientific, Waltham, MA, USA). Sequencing was performed using an Applied Biosystems 3730 DNA Analyzer with a 96-capillary array (Thermo Fisher Scientific, Waltham, MA, USA). BLASTN (E < 10^−4^) (Camacho et al., 2009) of BPMV inserts against Wm82.a2.v1 transcripts confirmed that silencing targets were restricted to the genes of interest and their homeologs (**Supplementary File 1**).

Williams 82 seeds were germinated in potting mix in separated 48-well insert trays. Trays were kept in growth chambers set to provide a 16-hour photoperiod at 24°C. Ten days after germination (VC growth stage; Fehr and Caviness, 1977), seedlings were bombarded with one of five target constructs (*GmRR4*, *GmGLU1*, *GmbHLH38*, *GmRR4*+*GmGLU1* [RG], and *GmbHLH38*+*GmGLU1* [HG]) or the empty vector (EV) construct, following the Whitham et al. (2016) protocol. Each construct was bombarded in triplicate. Three days after bombardment, the triplicate specimens were transplanted into a single 20-cm pot. Two weeks after transplant, positive plants were confirmed via ELISA, and leaf tissue with viral symptoms was collected, lyophilized, and stored at −20°C to serve as inoculum for subsequent experiments.

We hypothesized that silencing genes required for iron uptake and utilization would result in an IDC phenotype, even when plants were grown under iron-sufficient conditions. Therefore, seeds of iron-efficient Clark line (PI 548533) were germinated on paper at 24°C. Five days after germination (5 dag), seedlings were transferred to hydroponics. Eight 10-L buckets were set up with 18 seedlings in each bucket. All buckets were set up with iron-sufficient [100 µM Fe(NO_3_)_3_*9H_2_O] hydroponic solutions as described by Chaney et al. (1992), adapted for 10-L buckets. After full unifoliate emergence (10 dag; VC growth stage; Fehr and Caviness, 1977), seedlings were rub-inoculated with VIGS constructs generated as above, as described by Whitham et al. (2016). Four buckets were randomly assigned to each group of target genes [*GmRR4*+*GmGLU1* (RG group) or *GmbHLH38*+*GmGLU1* (HG group)]. Of the four buckets assigned to each group, two buckets were used for tissue collection for RNA-seq, and two buckets were used for phenotyping. Each group contained a control EV, two single-gene constructs, and a double-gene construct that contained both single-gene targets in the same construct. Four plants in each bucket were inoculated with one of the four constructs of the target gene group. Twelve days after inoculation (22 dag), tissue from the first trifoliolate and whole root tissue were harvested, frozen in liquid nitrogen, and then maintained at −80°C. Tissues were collected only from plants with visual viral symptoms.

### 2.2 Phenotype analyses

Soil plant analysis development (SPAD; Spectrum Technologies, Aurora, IL, USA) readings for the first and second trifoliolates were collected 20 days after inoculation. SPAD measurements have been used to map IDC QTLs and confirm the identification of IDC QTLs visual scores (Lin et al., 1997, Lin et al., 2000; Assefa et al., 2020). In addition, SPAD measurements have been used in IDC gene expression analyses (O’Rourke et al., 2007, O’Rourke et al., 2009; Atencio et al., 2021; Kohlhase et al., 2021) and to phenotype VIGS plants in terms of response to iron stress (Atwood et al., 2014; O’Rourke et al., 2021). The average of six SPAD readings per trifoliolate, two readings per leaflet, was calculated for the first and second trifoliolates for eight plants per construct. The data were analyzed using ASReml-R^2^ with a randomized complete block design with subsampling:


$$y_{ijk}=\mu +\beta_{i}+\tau_{j}+\epsilon_{ij}+\varepsilon_{ijk},$$

where *µ* is the overall mean, *β_i_* is the *i*th bucket, *τ_j_* is the *j*th VIGS construct, *ϵ_ij_* is the plot-level experimental error, and *ε_ijk_* is the effect of plant *k* within plot *ij*. Tukey’s honest significant difference (HSD) tests were used to compare best linear unbiased estimates (BLUEs) between each construct. Trifoliolates were analyzed separately.

### 2.3 RNA isolation and sequence analyses

RNA was extracted following the RNeasy^®^ Plant Mini Kit (Qiagen, Germantown, MD, USA) protocol. Extracted RNA was DNase treated in 50-μl reactions using the Ambion^®^ TURBO DNA-free™ Kit (Thermo Fisher Scientific, Waltham, MA, USA) and further purified using an RNeasy^®^ MinElute^®^ Cleanup Kit (Qiagen, Germantown, MD, USA). Final RNA concentration and quality were measured using an ND-1000 NanoDrop™ Spectrophotometer (Thermo Fisher Scientific, Waltham, MA, USA).

RNA samples from four biological replicates were sent to the Iowa State University DNA Facility. Prior to sequencing, the DNA facility validated the quality of each RNA sample using an Agilent^®^ 2100 Bioanalyzer™ (Agilent^®^, Santa Clara, CA, USA). This corresponded to 64 RNA samples (2 gene groups × 4 constructs × 4 replicates × 2 tissues). Library preparation was performed with 700 ng of total RNA per sample using the NEBNext Ultra II RNA Library Prep Kit for Illumina (New England Biolabs, Inc., Ipswich, MA, USA) following the manufacturer’s protocol. Sequences were generated on the Illumina NovaSeq 6000 platform (Illumina, Inc., San Diego, CA, USA) at the Iowa State University DNA Facility. Sixty-four samples were run on a single lane of the S2 flow cell using 100-cycle single-read sequencing. Raw fastq files and processed BAM files generated by this study were deposited in the National Center for Biotechnology Information Sequence Read Archive (NCBI SRA BioProject accession PRJNA777456).

File quality was checked prior to processing using FastQC^3^. To confirm VIGS infection in each sample, we used the program FastQ Screen (Wingett and Andrews, 2018), comparing reads from each sample to the soybean genome ( (Schmutz et al., 2010), *G. max* Wm82.a2.v1, Phytozome 12, 6/27/2018) and the sequence of RNA1 and RNA2 from the BPMV isolate used to develop the BPMV VIGS vector (GenBank Accessions GQ996949 and GQ996952). Scythe^4^, fastx_trimmer^5^, and Sickle^6^ were used to remove sequencing adaptors, barcodes, and bases with quality scores below 20. Cleaned fastq files were sorted and mapped to the soybean reference genome (see above) using TopHat2 (version 2.1.1; Kim et al., 2013). SAMtools (version 1.6; Danecek et al., 2021) was used to filter and sort reliably mapping reads.

### 2.4 Identification of genes differentially expressed in response to candidate gene silencing

BAM files were loaded into RStudio^7^,^8^, and the edgeR package (Robinson et al., 2010) was used to identify DEGs. Since the VIGS vectors contained fragments of the target genes, sequencing of viral RNAs would lead to inflated gene counts of the target genes and their homeologs. Therefore, target genes and their homeologs were removed from the count table prior to data normalization. While generating sequence reads, the ISU DNA Facility generated two technical replications of each sample. Counts were averaged across the two technical replications within each sample. Genes with counts per million (cpm) of one or more (cpm ≥ 1) in at least three samples were considered expressed and used for further analyses. Library sizes were normalized across samples within the target gene group × tissue type using the trimmed mean of M-values (TMM) method (Robinson and Oshlack, 2010). Comparisons were made between silenced plants and plants treated with the EV control, within tissue type and within target gene group. Genes with a false discovery rate (FDR) <0.05 were considered significantly differentially expressed.

### 2.5 Gene annotation and GO term enrichment of DEGs

All DEGs were annotated using the Gene Annotation Lookup^9^ tool under the SoyBase Tools tab (Grant et al., 2010). A Fisher’s exact test (Fisher, 1966) with Bonferroni correction (corrected p-value < 0.05; Bonferroni, 1935) was used to test for enriched gene ontology (GO) terms associated with a DEG list of interest compared to all genes in the soybean genome. To identify transcription factors within our DEGs, we took advantage of the SoyDB Transcription factor database (Wang et al., 2010). The SoyBase Gene Model Correspondence Lookup^10^ was used to update transcription factors from the SoyDB transcription factor database to *G. max* Wm82.a2.v1 gene calls. This information is presented in the **Supplementary Tables**.

### 2.6 Identification of regions syntenic to the Gm03 IDC QTL in soybean and *Arabidopsis thaliana*

Previously, Assefa et al. (2020) identified a region on Gm03 that contained 16 significant single-nucleotide polymorphisms (SNPs) associated with IDC tolerance and overlapped the known IDC QTL on Gm03. We used the SoyBase Genome Browser^11^ to query the SNP names against *G. max* Wm82.a2.v1 and identified the corresponding region (Gm03: 34,241,291 to 34,883,065). We added 100,000 bases on either side to facilitate the identification of syntenic regions. This 842-kilobase (kb) region (now Gm03: 34,141,291 to 34,983,065) was split into 5-kb fragments used to query the soybean (*G. max* Wm82.a2.v1) and *Arabidopsis* genomes (TAIRv10; Lamesch et al., 2012) using BLAST (Camacho et al., 2009). BLASTN (E < 10^−30^) against *G. max* Wm82.a2.v1 identified a known homeologous region on Gm19 (Gm19: 38,907,028 to 39,785,922). TBLASTX (E < 10^−30^) against TAIRv10 identified three syntenic regions: Chr2: 17,159,104 to 17,357,598; Chr3: 21,079,957 to 21,228,475; and Chr5: 1,121,828 to 1, 149,284. Based on the synteny between soybean and *Arabidopsis*, the Gm03 interval was adjusted to Gm03: 34,093,913 to 34,997,169.

To compare gene content between Gm03 and the other syntenic intervals, proteins from the Gm03 region (Glyma.03G126900 to Glyma.03G134600) were compared to all predicted proteins in the other intervals (Glyma.19G126900 to Glyma.19G136500, At2G41170 to At2G41630, At3G56960 to At3G57370, and At5G04130 to At5G04180) using BLASTP (Camacho et al., 2009). To confirm that no additional syntenic regions were present in soybean or *Arabidopsis*, the protein sequences from Glyma.03G126900 to Glyma.03G134600 were compared against all predicted proteins in soybean and *Arabidopsis* using BLASTP (E < 10^−4^). No additional regions were identified. To aid in visualization, soybean proteins with no BLASTP (E < 10^−10^) hits to any proteins in the *Arabidopsis* genome were removed. Similarly, soybean and *Arabidopsis* genes that were non-protein coding were removed.

## 3 Results

### 3.1 Phenotypic evaluation

**Figure 1:**
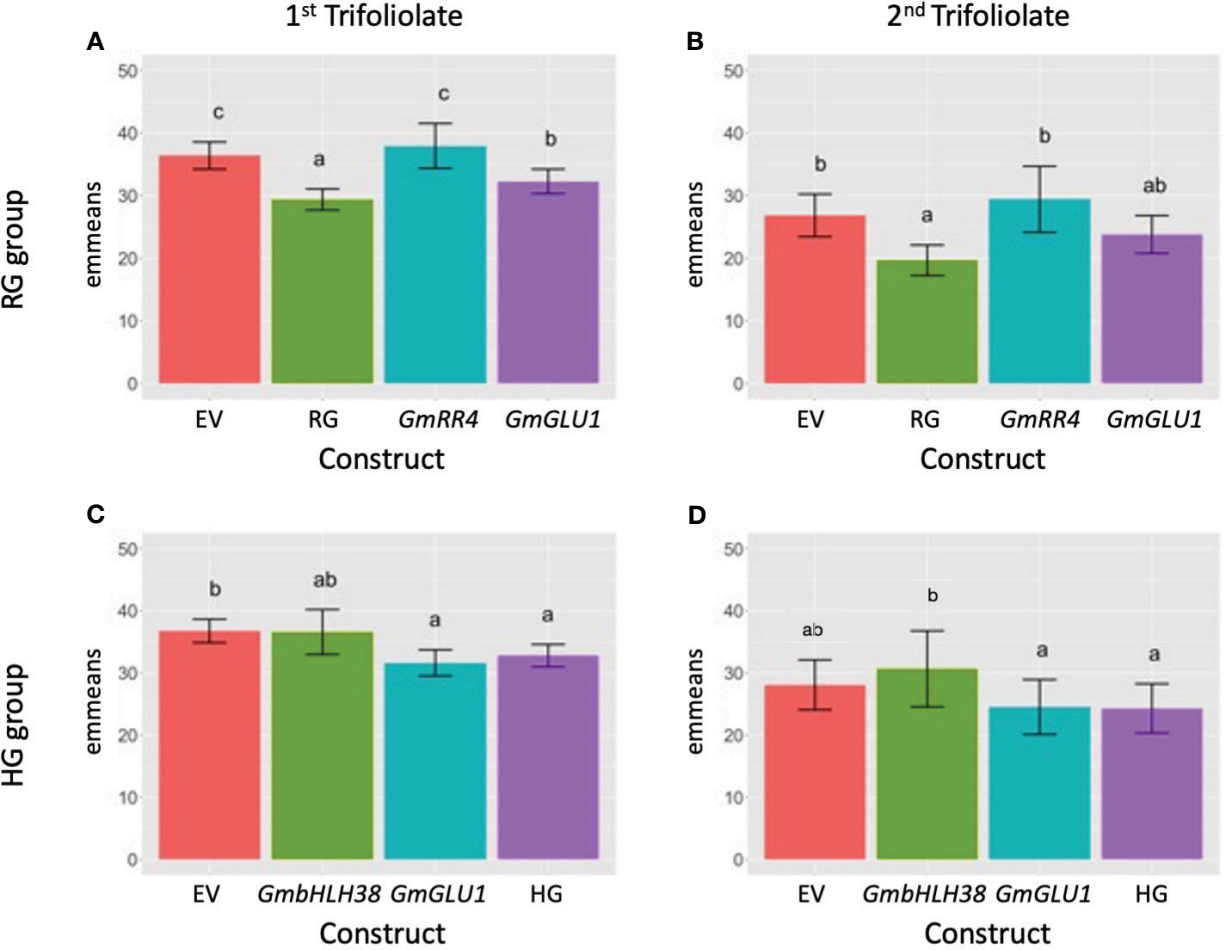
Effect of VIGS on leaf SPAD measurements. **(A, B)** BPMV-VIGS vectors were built with inserts targeting *GmRR4* (Glyma.03G128300), *GmGLU1* (Glyma.03G130000), and *GmRR4*+*GmGLU1* (RG). **(C, D)** BPMV-VIGS vectors were built with inserts targeting *GmbHLH38* (Glyma.03G130400, Glyma.03G130600), *GmGLU1* (Glyma.03G130000), and *GmbHLH38*+*GmGLU1* (HG). An empty vector (EV) control is included in each panel **(A–D)**. Construct names are provided beneath each bar in each panel. Clark (iron-efficient genotype) seedlings were inoculated 10 days after germination, and SPAD readings were collected on the first (panels **A, C**) and second (panels **B, D**) trifoliolate 20 days after inoculation. Plants were grown in iron-sufficient conditions. A randomized complete block design with subsampling was used to analyze the data. Tukey’s honest significant difference (HSD) tests were used to compare constructs within each group × tissue type. Letters indicate significant (alpha < 0.05) differences between constructs. VIGS, virus-induced gene silencing; SPAD, soil plant analysis development; BPMV, *bean pod mottle virus*.

SPAD measurements were collected from the first and second trifoliolates 20 days post-inoculation. Tukey’s HSD was used to calculate differences in emmeans among contrast treatment groups (**Figure 1**). We hypothesized that silencing one or more of the three candidate genes would impact the ability of Clark to take up and/or transport iron, leading to iron deficiency chlorosis (measured in the form of decreased SPAD readings), even when grown in iron-sufficient conditions.

For the RG group, we found significant differences between plants treated with different silencing constructs in both trifoliolates. In the first trifoliolate, *GmGLU1* had significantly lower SPAD values than the EV control and *GmRR4* (**Figure 1A**). The double-gene construct RG (*GmRR4* + *GmGLU1*) had significantly lower SPAD values than all other constructs, suggesting an additive effect between *GmGLU1* and *GmRR4*. In the second trifoliolate, there was no significant difference between *GmGLU1* and any of the other silencing constructs (**Figure 1B**). However, the SPAD values of RG remained significantly different than those of the empty vector control and *GmRR4*-silenced plants, supporting the idea of an additive effect of the targeted genes; both constructs needed to be silenced to make a measurable difference in the SPAD readings.

In the first trifoliolate of the HG group, none of the target gene constructs were significantly different from each other (**Figure 1C**). However, SPAD readings for plants treated with *GmGLU1* and the double-gene construct HG (*GmbHLH38*+*GmGLU1*) were significantly different from those of plants treated with the empty vector control. The substitution of *GmbHLH38* for *GmRR4* in the double construct removed the additive gene effect that we saw in *GmRR4* + *GmGLU1* SPAD readings from the RG groups. This suggests that *GmRR4* and *GmbHLH38* could have antagonistic effects or could be inversely regulated. Validation of these findings will be the basis of future experiments. In the second trifoliolate, we observed lower SPAD readings for *GmGLU1* and HG but did not find any significant differences between any of the constructs (**Figure 1D**).

### 3.2 Sequencing and infection summary

Sequence reads were generated from RNA from leaf and root tissue infected with each BPMV-VIGS construct. We used FastQ Screen (Wingett and Andrews, 2018) to validate BPMV infection. Across group × tissue type, we found 47.1% to 92.5% of reads mapped to BPMV, confirming BPMV infection in all samples submitted for sequencing (**Table 1**, **Supplementary File 3**). The empty vector control, with no gene of interest inserted, had the highest infection rate. Double constructs, targeting two genes of interest, had the lowest infection rate regardless of group × tissue type. This suggests an inverse relationship between target insert length and infection rate. In addition, leaves had higher infection rates (ranging from 72% to 92%) than roots (ranging from 47% to 77%) for all constructs. These results are consistent with those of Juvale et al. (2012), who observed weaker gene silencing in roots compared to leaves when using BPMV-VIGS to silence green fluorescent protein (GFP) in hairy roots. Infection rates of the HG construct in leaves and roots and the bHLH38 construct in leaves showed the greatest variation.

**Table 1:** Summary of BPMV-VIGS infection rates in soybean.

Group	Tissue	Construct	Average percentage mapped to BPMV	Standard deviation
RG	Leaves	EV	92.5	2.6
		*GmGLU1*	85.5	1.0
		*GmRR4*	81.0	1.4
		RG	77.4	2.2
	Roots	EV	74.2	3.0
		*GmGLU1*	63.6	7.3
		*GmRR4*	58.8	1.3
		RG	52.9	3.8
HG	Leaves	EV	92.2	1.7
		*GmbHLH38*	74.2	13.2
		*GmGLU1*	84.8	1.7
		HG	72.4	12.8
	Roots	EV	77.7	4.0
		*GmbHLH38*	55.5	4.5
		*GmGLU1*	65.6	1.7
		HG	47.1	13.8

BPMV, bean pod mottle virus; VIGS, virus-induced gene silencing; EV, empty vector.

After examination of read quality with FastQC and sample quality with bigPint (Rutter et al., 2019; Rutter and Cook, 2020), four samples from the RG group (three from leaves and one from roots) and three samples from the HG group (two from leaves and one from roots) were removed. At least three biological replicates per sample remained regardless of group or tissue type (**Supplementary File 2**). Raw count tables were generated for each construct group × tissue type. As expected, we found a disproportionate number of reads for the respective target genes and homeologs due to target gene fragments in the viral reads. We removed the target genes and homeologs from the count tables to prevent the disproportionate read counts from affecting normalization and subsequent analyses.

### 3.3 Differential expression

**Figure 2:**
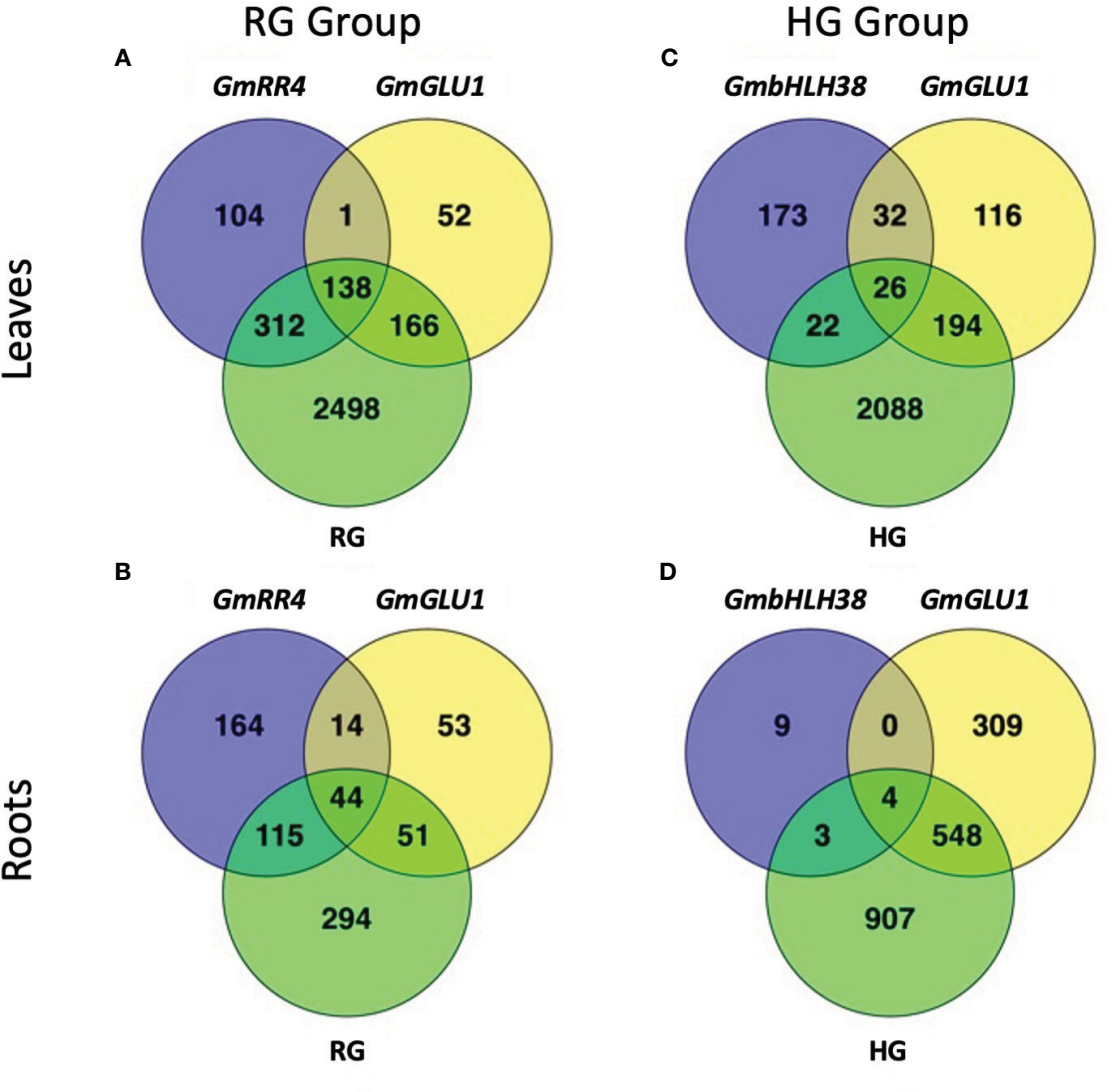
Number of overlapping differentially expressed genes between silencing constructs. BPMV-VIGS vectors were built with inserts targeting *GmRR4* (Glyma.03G128300), *GmGLU1* (Glyma.03G130000), and *GmbHLH38* (Glyma.03G130400, Glyma.03G130600). Two additional constructs were built to simultaneously target *GmRR4*+*GmGLU1* (RG) and *GmbHLH38*+*GmGLU1* (HG). Clark (iron-efficient genotype) seedlings were inoculated 10 days after germination, and tissue was collected 12 days after inoculation. Significant DEGs (FDR < 0.05) were identified between target genes and the empty vector control (Target − Control). DEG lists were compared within each construct group × tissue type **(A–D)**. BPMV, *bean pod mottle virus*; VIGS, virus-induced gene silencing; DEGs, differentially expressed genes; FDR, false discovery rate.

To examine the effect of each target gene construct, we compared gene expression relative to the empty vector control (EV-VIGS construct, **Supplementary File 2**). Since buckets were set up by group, each group had its own *GmGLU1* and empty vector control plants. Hereafter, we refer to each comparison by the name of the target gene. In leaves of the RG group, we found 357, 555, and 3,114 DEGs in *GmGLU1*, *GmRR4*, and RG plants, respectively. Between all three comparisons, we identified 138 common DEGs (**Figure 2A**). Four genes (Glyma.03G132700, Glyma.03G187700, Glyma.03G219200, and Glyma.03G228900) were from introgressed regions between near-isogenic soybean lines, Clark and IsoClark (Severin et al., 2010; Stec et al., 2013), including one from the narrowed Gm03 IDC QTL defined by Assefa et al. (2020). In the roots of the RG group plants, we found 162, 337, and 504 DEGs in *GmGLU1*, *GmRR4*, and RG plants, respectively. There were 44 DEGs common to all three constructs (**Figure 2B**).

In the leaves of the HG group, we found 253, 368, and 2,330 DEGs in *GmbHLH38*, *GmGLU1*, and HG, respectively. There were 26 DEGs common to the three constructs (**Figure 2C**). In the roots of the HG group, we found 16, 861, and 1,462 DEGs in *GmbHLH38*, *GmGLU1*, and HG plants, respectively. Surprisingly, *GmbHLH38* had very few DEGs, but we still identified four DEGs that were common to all three constructs (**Figure 2D**). One gene in common (Glyma.08G330100) was from an introgressed region between Clark and IsoClark (Severin et al., 2010; Stec et al., 2013).

**Figure 3:**
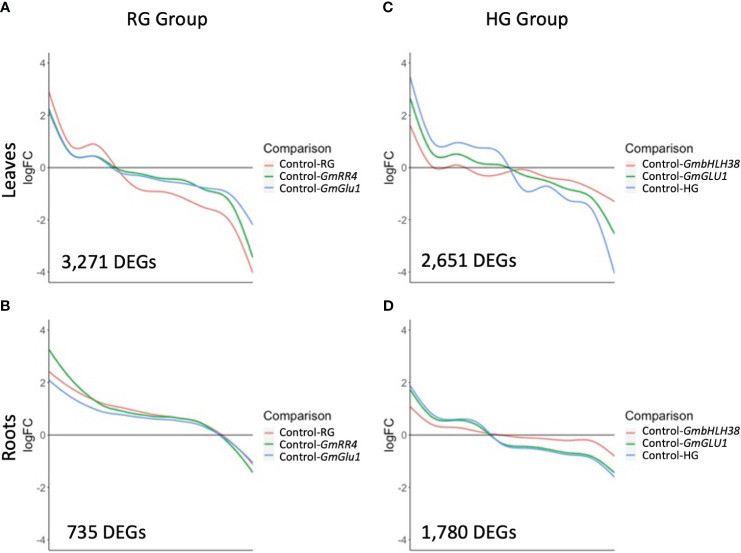
Expression trends of differentially expressed genes (DEGs) responding to silencing. BPMV-VIGS vectors were built with inserts targeting *GmRR4* (Glyma.03G128300), *GmGLU1* (Glyma.03G130000), and *GmbHLH38* (Glyma.03G130400, Glyma.03G130600). Two additional constructs were built to simultaneously target *GmRR4*+*GmGLU1* (RG) and *GmbHLH38*+*GmGLU1* (HG). Clark (iron-efficient genotype) seedlings were inoculated 10 days after germination, and tissue was collected 12 days after inoculation. Significant DEGs (FDR < 0.05) were identified between target genes and the empty vector control (Target/Control). Log_2_FC was plotted for all significant DEGs identified within a construct group × tissue type combination. **(A–D)** Smoothed conditional means were used to draw trend lines across DEGs for each construct. *BPMV*, bean pod mottle virus; VIGS, virus-induced gene silencing; FDR, false discovery rate.

**Figure 4:**
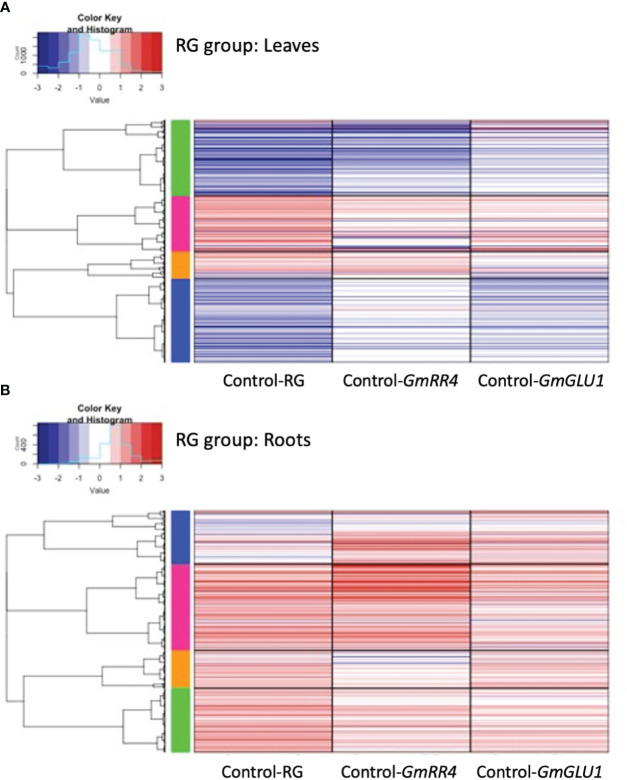
Heatmaps of differentially expressed genes (DEGs) responding to silencing in the RG group. BPMV-VIGS vectors were built with inserts targeting *GmRR4* (Glyma.03G128300) and *GmGLU1* (Glyma.03G130000) individually and *GmRR4*+*GmGLU1* simultaneously (RG). Clark (iron-efficient genotype) seedlings were inoculated 10 days after germination, and tissue was collected from leaves **(A)** and roots **(B)** 12 days after inoculation. Significant DEGs (FDR < 0.05) were identified between target genes and the empty vector control (Target/Control). FC was plotted for each significant DEG identified within a construct group × tissue type combination. Induced and repressed genes are depicted as red and blue bars, respectively; color intensity indicates the magnitude of the log_2_FC. *BPMV*, bean pod mottle virus; VIGS, virus-induced gene silencing; FDR, false discovery rate.

**Figure 5:**
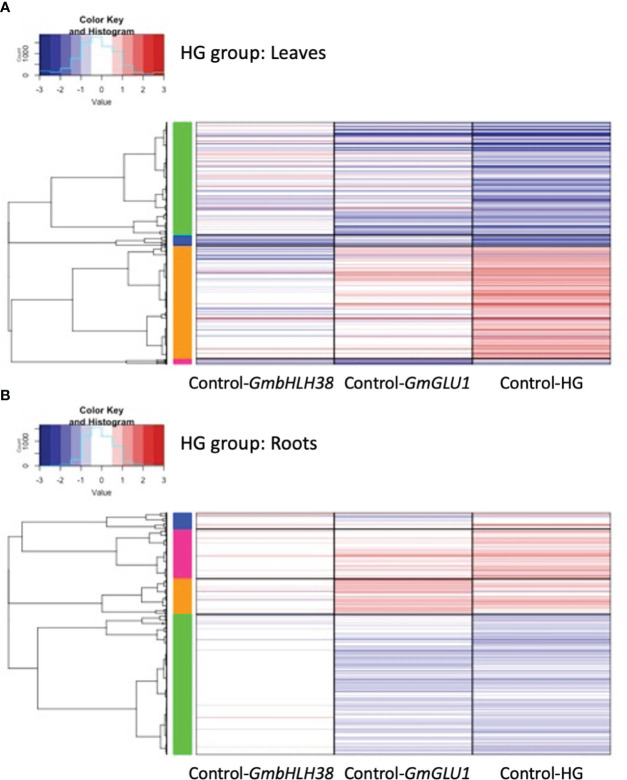
Heatmaps of differentially expressed genes (DEGs) responding to silencing in the HG group. BPMV-VIGS vectors were built with inserts targeting *GmbHLH38* (Glyma.03G130400, Glyma.03G130600) and *GmGLU1* (Glyma.03G130000) individually, and*GmbHLH38*+*GmGLU1* simultaneously (HG). Clark (iron-efficient genotype) seedlings were inoculated 10 days after germination, and tissue was collected from leaves **(A)** and roots **(B)** 12 days after inoculation. Significant DEGs (FDR < 0.05) were identified between target genes and the empty control vector (Target/Control). FC was plotted for each significant DEG identified within a construct group × tissue type combination. Induced and repressed genes are depicted as red and blue bars, respectively; color intensity indicates the magnitude of the log_2_FC. *BPMV*, bean pod mottle virus; VIGS, virus-induced gene silencing; FDR, false discovery rate.

To gain insight into expression trends in DEGs from each group, we plotted the log_2_ fold change (log_2_FC) for the 3,271 and 735 unique DEGs identified from leaves and roots, respectively, of the RG group and the 2,651 and 1,780 unique DEGs identified from leaves and roots, respectively, of the HG group (**Figure 3**, **Supplementary File 3**). We then used hierarchical clustering to generate groups of genes with similar expression patterns, which were visualized via heatmaps (**Figures 4**, **5**, **Supplementary File 4**).

### 3.4 RG group

Plotting log_2_FC across the 3,271 RG group DEGs in leaves revealed that the absolute log_2_FC of the double construct was greater than that of the two single constructs (**Figure 3A**). Interestingly, *GmRR4* and *GmGLU1* had very similar log_2_FC values in upregulated genes but were more distinct in downregulated genes. Nevertheless, the direction of regulation (up- or downregulated) across DEGs was consistent for all constructs. This suggests that silencing both target genes simultaneously resulted in greater expression changes than silencing the individual genes. In fact, for 1,552 DEGs (47.4%), the absolute log_2_FC of the double construct was greater than the additive effect of both single-gene constructs. Among the 735 DEGs from roots, log_2_FC patterns were almost identical between all three constructs except for a small group of genes with greatest differential expression in *GmRR4* (**Figure 3B**). Unlike the leaves, only 87 DEGs (11.8%) had an absolute log_2_FC for the double construct greater than the additive effect of both single-gene constructs.

These same expression patterns can be observed in more detail in **Figure 4**. Clustering of the 3,271 RG-group leaf DEGs separated them into four gene clusters (**Figure 4A**). The green cluster was primarily downregulated in RG and *GmRR4* and weakly downregulated in *GmGLU1*. The green cluster contained 1,017 DEGs and 26 significantly overrepresented GO terms (**Supplementary File 4**), which were associated with hormone signaling, responses and biosynthesis (abscisic acid (ABA)-mediated signaling, jasmonic acid (JA) biosynthesis, and response to JA stimulus and ethylene), defense (anthocyanin-containing compound, chalcone, flavonoid, lignin and lignan biosynthesis, defense response to bacterium, incompatible interaction, and response to fungus and other organisms), and stress (response to gravity, oxidative stress, UV, UV-B, and wounding). In contrast, the blue cluster, containing 1,131 DEGs and 42 significantly overrepresented GO terms, was downregulated in RG and *GmGLU1* and weakly downregulated in *GmRR4*. While the blue cluster was also associated with hormone signaling, defense, and stress responses, only two GO terms were common to the green and blue clusters (response to other organisms and defense response, incompatible interaction). Hormone-associated GO terms in the blue cluster included induced systemic acquired resistance (SAR), salicylic acid (SA)-mediated signaling, SA biosynthesis, induced SAR, and JA-mediated signaling. Defense GO terms included defense response to bacteria and fungi, innate immune response, and MAPK cascade, among many others. In addition, we also found evidence of potential nutrient stress, including amino acid transport, ammonium transport, and cellular response to nitrogen starvation. The pink cluster contained 759 DEGs and three significant GO terms associated with energy and gene silencing (generation of precursor metabolites and energy, production of siRNA involved in RNA interference, and virus-induced gene silencing). The orange cluster contained 364 DEGs and four significant GO terms associated with photosynthesis and energy (photosystem II assembly, thylakoid membrane organization, generation of precursor metabolites and energy, and photosynthesis). The difference between the pink and orange clusters was the contribution of *GmGLU1* and *GmRR4*, as observed for the blue and green clusters.

As in the leaves, the 735 DEGs from the roots of the RG group separated into four expression clusters (**Figure 4B**). In contrast to the leaves, we saw minimal differences in expression patterns between constructs in roots; most genes were upregulated relative to the empty vector control. The blue cluster contained 162 DEGs and nine significant GO terms associated with the cell wall (cell wall, plant-type cell wall, secondary cell wall biogenesis, cell wall macromolecule, glucuronoxylan, rhamnogalacturonan I side chain metabolic metabolism, lignin and xylan biosynthesis, and lignin catabolism). The pink cluster contained 263 DEGs and two significant GO terms associated with plant hormones (regulation of salicylic acid and brassinosteroid biosynthesis) and two terms associated with defense (defense response and systemic acquired resistance). The green cluster contained 195 DEGs and a single overrepresented GO term, protein retention in the ER lumen. No significant GO terms were associated with the 298 DEGs in the orange cluster.

### 3.5 HG group

Expression patterns of 2,651 DEGs in the leaves of the HG group showed similarities to those of the RG group; the double construct had greater absolute log_2_FC values than both single constructs, and the direction of gene regulation was similar for all three constructs (**Figure 3C**). One striking difference in the HG group was the greater distinction of log_2_FC values between the three constructs. For 1,633 (61.5%) DEGs, the absolute log_2_FC of HG was greater than the additive effect of the single-gene constructs. While many genes in *GmbHLH38* had small log_2_FC values, there still appeared to be an additive effect with *GmGLU1* on HG log_2_FC values. In the roots of the HG group (**Figure 3D**), we saw significant overlap between the HG and *GmGLU1* log_2_FC values. For 463 (25.9%) of the 1781 DEGs, the absolute log_2_FC of HG was greater than the additive effect of the single-gene constructs. The DEGs from the *GmbHLH38* construct always had lower log_2_FC values compared to the other two constructs. This suggests that silencing of *GmbHLH38* had a positive effect in the case of the double construct in leaves but had a negligible effect in roots.

Four gene clusters were identified in the heatmap of the 2651 leaf DEGs from the HG group (**Figure 5A**). The HG construct had stronger expression changes across DEGs than either of the single constructs. The green and orange clusters contained 93% of the DEGs and were generally down- and upregulated, respectively. The green cluster contained 1,237 DEGs and 46 significant GO terms associated with hormone signaling, defense, and stress responses (**Supplementary File 4**). Of the 46 significant GO terms, 23 were also significant in the blue cluster from the RG group. The orange cluster contained 1,232 DEGs and 53 overrepresented GO terms associated with photosynthesis, energy and cation homeostasis, and transport (**Supplementary File 4**). Photosynthesis-related GO terms included response to light, photosynthetic electron transport, and photosystem II assembly, repair, and stabilization. Energy-associated GO terms included ATP synthesis coupled proton transport and generation of precursor metabolites and energy. Cation-related GO terms included cellular cation homeostasis and divalent metal ion transport. Surprisingly, the orange cluster was the first to have an overrepresented GO term directly related to iron (iron–sulfur cluster assembly). No significant GO terms were associated with the blue and pink clusters.

Four gene clusters were identified in the heatmap from the 1,780 DEGs of the HG group in roots (**Figure 5B**). The DEGs from the *GmbHLH38* construct showed little to no expression changes in the majority of the genes. HG and GLU1 had very similar expression patterns across DEGs, with greater expression observed in HG. Genes in the blue, pink, and orange clusters were induced, while genes in the green cluster were repressed. The blue cluster contained 122 DEGs and a single significant GO term, secondary cell wall biogenesis. The pink cluster contained 364 DEGs and 18 significant GO terms associated with the cell wall (cell wall, primary cell wall, secondary cell wall, plant-type cell wall biogenesis, cellulose biosynthesis, and cellulose metabolism) and growth (developmental programmed cell death, regulation of cell size, and multidimensional cell growth). The green cluster contained 1,032 DEGs and 24 significant GO terms, mainly associated with photosynthesis (**Supplementary File 4**). Of these, 21 were in common with the orange cluster from the leaves of the HG group. While these GO terms were induced in HG leaves, they were repressed in HG roots.

### 3.6 Characterizing regions syntenic to the IDC QTL on Gm03 in soybean and *Arabidopsis*

**Figure 6:**
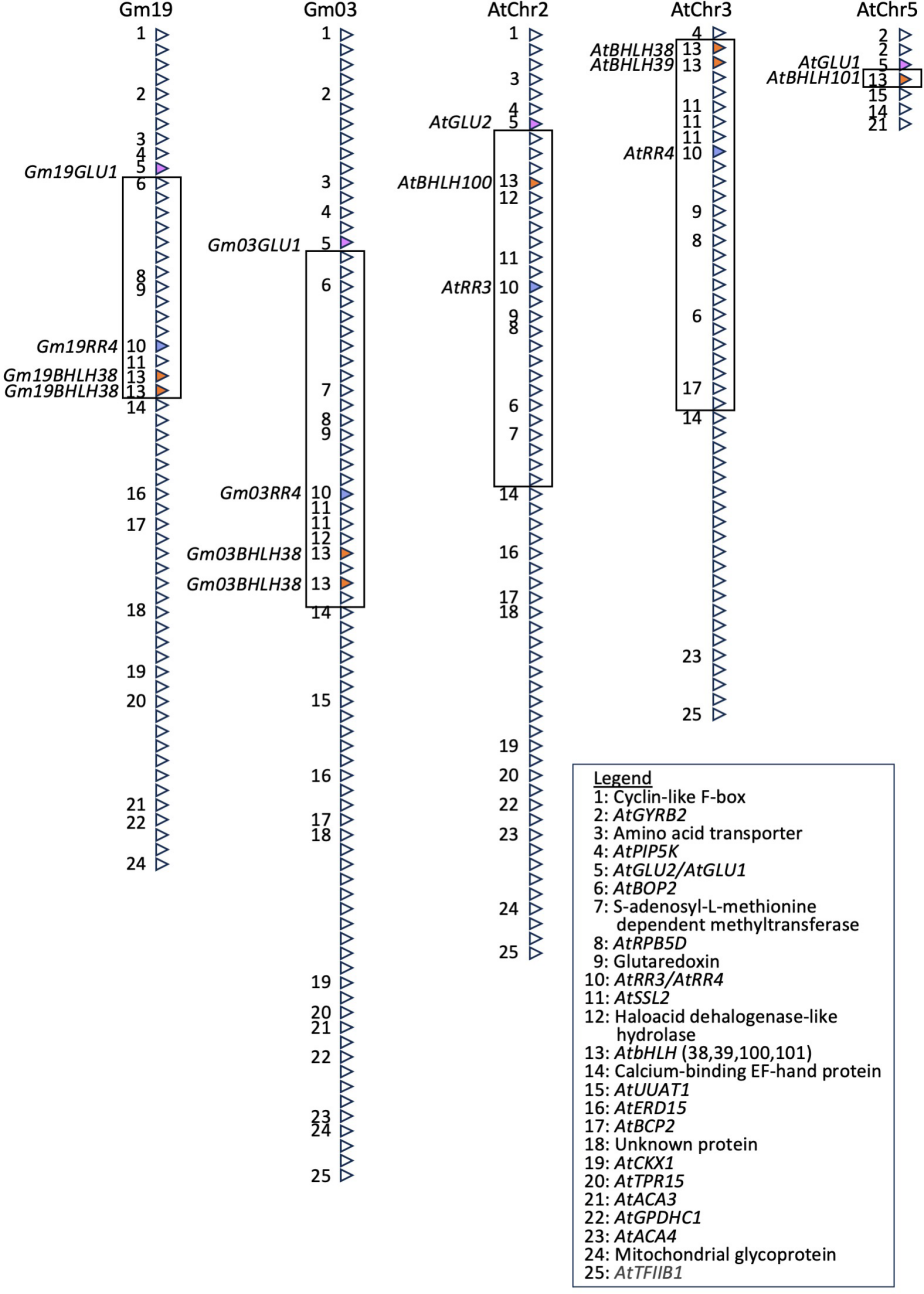
The Gm03 IDC QTL is syntenic to regions on Gm19, AtChr2, AtChr3, and AtChr5 and corresponds to known iron stress genes, including *AtbHLH38*, *AtbHLH39*, *AtbHLH100*, *AtbHLH101*, and *AtGLU1*. A series of BLAST searches (Camacho et al., 2009) were used to identify regions homeologous or syntenic to the Gm03 IDC QTL in soybean. Genes with no homology to *Arabidopsis* and non-protein coding genes have been removed. Similarly, spaces between genes and gene orientation have also been removed. Only genes conserved between Gm03 and at least one other region are labeled. Homologs of *AtGLU1*, *AtbHLH* transcription factors (38, 39, 100, and 101), and *AtRR4* are colored pink, dark orange, and blue, respectively. IDC, iron deficiency chlorosis; QTL, quantitative trait locus.

Given the failure of the *GmBHLH38* construct to induce IDC symptom development or differential expression of iron-related genes, we needed to reassess its predicted function relative to *Arabidopsis*. Rather than relying only on sequence homology between bHLHs, we needed to determine whether there was a region syntenic to the Gm03 IDC QTL in *Arabidopsis*. We used the 730-kb region identified by Assefa et al. (2020) as a starting point for our analyses. This region contains four distinct linkage blocks, each hypothesized to contain a candidate IDC candidate gene. In addition, this region spans the narrowed IDC introgression identified by Peiffer et al. (2012) and is also within the known introgressed region on Gm03 (Severin et al., 2010; Stec et al., 2013). Using a series of BLAST analyses (Camacho et al., 2009), we were able to identify the homeologous region on soybean chromosome 19 and syntenic regions on *Arabidopsis* AtChr2, AtChr3, and AtChr5 (**Figure 6**). To simplify **Figure 6**, only protein-coding genes found in the chromosome 3 QTL and in at least one other syntenic location are annotated. In addition, all spaces between genes have been removed.

Looking at **Figure 6**, it is evident that there has been an inversion in *Arabidopsis*, relative to the Gm03 and Gm19 regions. This inversion includes genes labeled 6–13 (*GmBop2* to *GmbHLH38*) on Gm03 and Gm19, genes 6–13 on AtChr2 (*AtBop2* to *AtbHLH100*), and genes 6, 8–11, and 13 (*AtBop2* to *AtbHLH38/39*) on AtChr3. Almost the entire inverted region has been lost on AtChr5, except for gene 13, which corresponds to *AtbHLH101*. While we were unable to determine where the inversion occurred between genes 5 and 6 and genes 13 and 14, this inversion would suggest that the *AtbHLH38*, *AtbHLH39*, *AtbHLH100*, and *AtbHLH101* genes in *Arabidopsis* are in a different genomic environment compared to the *Gm03bHLH38* and *Gm19bHLH38* genes in soybean. Further, **Figure 6** demonstrates that soybean lacks *AtBHLH100* and *AtbHLH101* homeologs, as confirmed by BLASTP (E < 10^−12^) of *AtBHLH100* and *AtbHLH101* against all proteins in the soybean genome.

## 4 Discussion

Virus-induced gene silencing is a relatively quick method for testing candidate gene function. Coupled with RNA-seq, it can identify the global network of genes contributing to agronomically important phenotypes. In this study, we developed VIGS constructs to target four genes located within the historical IDC QTL on soybean chromosome Gm03. Previous data suggest that multiple genes within this QTL could confer IDC tolerance. Peiffer et al. (2012) narrowed the previously identified IDC QTL on Gm03 (Lin et al., 1997) to 250 kb by fine-mapping sub-near isogenic lines developed from Clark and IsoClark. This resulted in the identification of 18 candidate genes, including two homologs of *AtBHLH38* (Glyma.03G130400 and Glyma.03G130600, also evaluated in this study). Sequencing of these genes in iron-efficient and iron-inefficient lines revealed a 12-bp deletion in a Glyma.03g130400 specific to iron-inefficient lines. It is worth noting, however, that of the 18 candidate genes identified, eight were differentially expressed between Clark and IsoClark, suggesting the potential for additional candidate genes. The Assefa et al. (2020) GWA study identified 16 significant SNPs clustered across the IDC QTL on Gm03. An examination of linkage disequilibrium in this region identified four distinct linkage blocks, each thought to contain a candidate gene for IDC tolerance. The four genes targeted in this study correspond to three of the four linkage blocks identified by Assefa et al. (2020), the region introgressed from the iron-inefficient line T203 into iron-efficient Clark, leading to the development of iron-inefficient IsoClark (Severin et al., 2010; Stec et al., 2013), and the 250 kb narrowed introgression identified and characterized by Peiffer et al. (2012).

In this study, we developed BPMV constructs targeting *GmRR4* (Glyma.03G128300), *GmGLU1* (Glyma.03G130000), and *GmbHLH38* (Glyma.03G130400 and Glyma.03G130600). We hypothesized that silencing genes required for iron uptake and homeostasis would result in the development of IDC symptoms, even when plants were grown in iron-sufficient conditions (**Figure 1**). To understand how each of the silenced genes contributed to IDC symptom development, we conducted RNA-seq analyses of silenced plants representing each construct (**Figures 2**, **3**). Hierarchical clustering was used to generate groups of DEGs with similar expression patterns, and GO term enrichment was used to assign biological functions to each cluster (**Figures 4**, **5**).

The finding that *GmGLU1*/*GmRR4* plants were significantly different from the empty vector and *GmGLU1* plants suggested that both *GmGLU1* and *GmRR4* silencing impacted chlorotic symptom development. *GmRR4* was selected as a VIGS target because both Atwood et al. (2014) and Moran Lauter et al (Moran Lauter et al., 2014, Moran Lauter et al., 2020). observed differential expression of genes associated with the circadian rhythm, cell cycle, and defense in response to iron stress. Li et al. (2019) demonstrated that short-term iron stress modulates different circadian clock components in *Arabidopsis* and soybean. They hypothesized that changes in clock period and phase in soybean could allow more time for iron uptake during key biological processes, such as photosynthesis. *Arabidopsis AtRR4*, also known as *ARR9*, is regulated by the circadian clock and by cytokinin (Ishida et al., 2008). Seven different response regulators, including *AtRR4*, are repressed by the phytotoxin coronatine during infection by *Pseudomonas syringae* pv. *tomato* DC3000 (Thilmony et al., 2006). In the leaves, silencing of *GmRR4* had the largest effect on the green cluster (**Figure 4A**), which was associated with hormone signaling and biosynthesis, defense, and general stress responses. In the roots, *GmRR4* silencing had the most impact on the pink cluster (**Figure 4B**), associated with the regulation of salicylic acid, defense, and systemic acquired resistance.

Numerous studies have focused on *AtGLU1* in *Arabidopsis*. Kissen et al. (2010) compared the T-DNA mutant *glu1-2* with wild-type *Arabidopsis*. The mutant exhibited more chlorotic leaves relative to the wild type when grown in nutrient-sufficient conditions. Microarray analyses revealed extensive transcriptional reprogramming, including repression of photosynthesis, photorespiration, and chlorophyll biosynthesis, and induction of multiple stress responses (cold, heat, drought, and oxidative stress). In addition, genes associated with glutamate biosynthesis, amino acid biosynthesis, and nitrogen assimilation were also significantly impacted. We also saw evidence of the impact of *GmGLU1* silencing on nitrogen assimilation (GO terms: ammonium transport and cellular response to nitrogen starvation) and amino acid biosynthesis (GO terms: amino acid import and amino acid transport; **Supplementary File 4**). More recently, Cui et al. (2020) have described the *AtGLU1* mutant (*glu1-4*), which is associated with the development of light leaf chlorosis under normal conditions, but severe chlorosis and reduced iron content in the leaves under iron stress conditions. Interestingly, AtGLU1, AtGLU2, and glutamate transporter AtGLT1 have redundant functions in silencing transposable element activation under nitrogen starvation conditions (Wang et al., 2022), so it is possible that AtGLU2 and AtGLT1 may also function in iron stress responses.

In contrast, *GmGLU1/GmbHLH38* plants were not significantly different from *GmGLU1*, suggesting that *GmbHLH38* had no impact on IDC symptom development. Cui et al. (2020) found that the *glu1-4* mutant had reduced expression of several iron stress-responsive genes in the shoots, including *AtbHLH38*, *AtbHLH39*, *AtbHLH100*, and *AtbHLH101*. Based on this evidence, it is possible that *GmbHLH38* was already repressed by *GmGLU1* silencing, and no additional repression would be expected. However, the GmbHLH38 single-gene construct also had no visible phenotype. Based on the *Arabidopsis* literature, this is also not surprising, as Wang et al. (2007) found that single-insertion mutants of *AtbHLH38*, *AtbHLH39*, *AtbHLH100*, and *AtbHLH101* exhibited no change in phenotype and were able to induce normal iron stress responses, likely due to redundancy between genes. Only double knockouts of *AtbHLH39*/*AtbHLH100* and *AtbHLH39*/*AtbHLH101* were associated with visible phenotypes in iron-sufficient and iron-deficient conditions and had decreased iron content in the leaves, and only the triple knockout *AtbHLH39*/*AtbHLH100*/*AtbHLH101* developed lethal chlorotic symptoms under iron stress conditions (Wang et al., 2013). The authors concluded that all four genes played redundant roles in regulating iron stress responses. However, the impact on iron stress responses of knocking out each gene varied. Recently, Li et al. (2018) demonstrated that overexpression of *GmbHLH38* (identified as *GmbHLH300*, Glyma.03G130600) with an ortholog of *AtFIT* (*Glyma.12G178500*, identified as *GmbHLH57*) conferred enhanced tolerance to iron deficiency. If *GmbHLH38* is functional and soybean lacks homologs of *AtbHLH39*, *AtbHLH100*, and *AtbHLH101* as demonstrated in our synteny analyses, where does the functional redundancy suggested by our silencing of *GmbHLH38* come from? While the most likely candidates are the *GmbHLH38* homeologs on Gm19, these genes would also have been silenced by the *GmbHLH38* construct. Therefore, redundant genes, significantly different from *AtbHLH39*, *AtbHLH100*, and *AtbHLH101*, must exist elsewhere in the soybean genome, suggesting important differences in the regulation of iron stress responses between soybean and *Arabidopsis*.

To help in understanding why silencing *GmBHLH38* did not result in a visible phenotype or altered expression of iron uptake and homeostasis genes, we searched for synteny between the IDC QTL on Gm03 and *Arabidopsis*. We identified a single homeologous region on Gm19 and three syntenic regions on AtChr2, AtChr3, and AtChr5. These regions include *AtGLU1* and *AtGLU2*; *AtRR3* and *AtRR4*; and *AtbHLH38*, *AtbHLH39*, *AtbHLH100*, and *AtbHLH101*. The syntenic arrangement of the four AtbHLHs helps to explain how they are directly regulated by AtbHLH34, AtbHLH104 (Li et al., 2016), AtbHLH115 (Liang et al., 2017), and AtbHLH21 (Gao et al., 2020). Recently, Chen et al. (2021) have demonstrated that AtbHLH39, AtbHLH100, and AtbHLH101 are also negative regulators of flowering under long days in *Arabidopsis*. Direct interaction with CONSTANS (CO) represses the expression of *FLOWERING LOCUS T* (*FT*). Our results identify two additional genes contributing to iron stress tolerance within this region in soybean. This suggests that these genomic segments could be important in iron stress responses and regulation of flowering time across agronomically important plant species. Therefore, we were interested in determining whether synteny could be used as a “marker” for the identification of corresponding regions in other species. As proof of concept, we examined the genomic context of *fefe*, a bHLH38 transcription factor regulating iron uptake in melon (Ramamurthy and Waters, 2017). We used the gene corresponding to *fefe* (MELO3CO19065) to browse the surrounding region in the Melonomics genome browser^12^. From *MELO3CO19040* to *MELO3C019075*, we identified four additional genes (not bHLHs) corresponding to **Figure 6** (genes 4, 7, 9, 10), suggesting that this region in cucumber is syntenic to the IDC QTL on Gm03. Using the gene identifier of *Gm03bHLH38* (*Glyma*.03G130600) as a query term in the Legume Information System Genome Context Viewer^13^, we could easily identify the orthologous and homeologous regions across multiple species in the legume genera *Arachis*, *Cajanus*, *Cicer*, *Glycine*, *Lotus*, *Lupinus*, *Medicago*, *Phaseolus*, *Pisum*, *Trifolium*, and *Vigna*, which include agronomically important crop species such as peanut, chickpea, alfalfa, and common bean. Singh et al. (2023) conducted transcriptome analyses of two chickpea (*Cicer arietinum*) genotypes with high and low iron content in the seed. We examined their data for homeologs of *GmGLU1*, *GmRR4*, and *GmbHLH38*. *CabHLH38*, *CaGLU1*, and *CaRR4* were only differentially expressed in response to iron deficiency in the high-seed iron genotype. Given that few genes required for iron stress responses have been characterized and validated in legumes (Sharma et al., 2023), this finding suggests that this region can be used as a tool to identify candidate genes involved in iron stress responses conserved across a broad range of species.

In conclusion, these results provide valuable insight into the effects of *GmGLU1* and *GmRR4* on the soybean iron stress response. To our knowledge, this is the first demonstration of an IDC QTL conserved across multiple species and containing multiple genes conferring iron stress tolerance. This connection will enable the identification of candidate genes and networks underlying iron stress responses across a broad range of agronomically important crops.

## Data availability statement

The datasets presented in this study can be found in online repositories. The names of the repository/repositories and accession number(s) can be found below: https://www.ncbi.nlm.nih.gov/, PRJNA777456.

## Author contributions

DK: Conceptualization, Data curation, Formal analysis, Investigation, Methodology, Software, Writing – original draft, Writing – review & editing. JO’R: Conceptualization, Investigation, Writing – review & editing. MG: Conceptualization, Data curation, Formal analysis, Investigation, Methodology, Project administration, Resources, Software, Supervision, Validation, Visualization, Writing – original draft, Writing – review & editing.

## Footnotes

1 SoyStats 2023. http://soystats.com.

2 Butler, D.G. Cullis, B.R. Gilmour, A.R., Thompson, R. ASRemb-R Reference Manual Version 4.2. https://asreml.kb.vsni.co.uk/wp-content/uploads/sites/3/ASReml-R-Reference-Manual-4.2.pdf.

3 FastQC: a quality control tool for high throughput sequence data. Version 0.11.3. http://www.bioinformatics.babraham.ac.uk/projects/fastqc/.

4 Scythe- a Bayesian adapter trimmer. Version 0.981. https://github.com/vsbuffalo/scythe.

5 FASTX-Tookit. Version 0.0.14. http://hannonlab.cshl.edu/fastx_toolkit/.

6 Sickle- a windowed adaptive trimming tool for FASTQ files using quality. Version 1.2. https://github.com/najoshi/sickle/.

7 R Core Team. R: A language and environment for statistical computing. R Foundation for Statistical Computing, Vienna, Austria. https://www.R-project.org/.

8 RStudio: integrated development for R. http://www.rstudio.com/.

9 SoyBase Gene Annotation Tool. Version Wm82.a2.v1. https://www.soybase.org/genomeannotation/.

10 SoyBase Gene Model Correspondence Lookup. https://www.soybase.org/correspondence/.

11 SoyBase Genome Browser. Version Wm82.a2.v1. https://www.soybase.org/SequenceIntro.php/.

12 Melonomics Jbrowse. Melon version 4.0. https://www.melonomics.net/melonomics.html#/jbrowse.

13 Legume Information System Genome Context Viewer. https://gcv.legumeinfo.org/gcv2/instructions.
